# Improving the endoscopic recognition of early colorectal carcinoma using artificial intelligence: current evidence and future directions

**DOI:** 10.1055/a-2403-3103

**Published:** 2024-10-10

**Authors:** Ayla Thijssen, Ramon-Michel Schreuder, Nikoo Dehghani, Marieke Schor, Peter H.N. de With, Fons van der Sommen, Jurjen J. Boonstra, Leon M.G. Moons, Erik J. Schoon

**Affiliations:** 1 5211 GROW Research Institute for Oncology and Reproduction, Maastricht University, Maastricht, Netherlands; 2 199236 Department of Gastroenterology and Hepatology, Maastricht Universitair Medisch Centrum+, Maastricht, Netherlands; 3 3168 Department of Gastroenterology and Hepatology, Catharina Hospital, Eindhoven, Netherlands; 4 Department of Electrical Engineering, Eindhoven University of Technology, Eindhoven, Netherlands; 5 5211 University Library, Department of Education and Support, Maastricht University, Maastricht, Netherlands; 6 4501 Department of Gastroenterology and Hepatology, Leids Universitair Medisch Centrum, Leiden, Netherlands; 7 Department of Gastroenterology and Hepatology, University Medical Center Utrecht, Utrecht, Netherlands

**Keywords:** Endoscopy Lower GI Tract, Colorectal cancer, Polyps / adenomas / ..., Diagnosis and imaging (inc chromoendoscopy, NBI, iSCAN, FICE, CLE...), Quality and logistical aspects, Image and data processing, documentatiton

## Abstract

**Background and study aims** Artificial intelligence (AI) has great potential to improve endoscopic recognition of early stage colorectal carcinoma (CRC). This scoping review aimed to summarize current evidence on this topic, provide an overview of the methodologies currently used, and guide future research.

**Methods** A systematic search was performed following the PRISMA-Scr guideline. PubMed (including Medline), Scopus, Embase, IEEE Xplore, and ACM Digital Library were searched up to January 2024. Studies were eligible for inclusion when using AI for distinguishing CRC from colorectal polyps on endoscopic imaging, using histopathology as gold standard, reporting sensitivity, specificity, or accuracy as outcomes.

**Results** Of 5024 screened articles, 26 were included. Computer-aided diagnosis (CADx) system classification categories ranged from two categories, such as lesions suitable or unsuitable for endoscopic resection, to five categories, such as hyperplastic polyp, sessile serrated lesion, adenoma, cancer, and other. The number of images used in testing databases varied from 69 to 84,585. Diagnostic performances were divergent, with sensitivities varying from 55.0% to 99.2%, specificities from 67.5% to 100% and accuracies from 74.4% to 94.4%.

**Conclusions** This review highlights that using AI to improve endoscopic recognition of early stage CRC is an upcoming research field. We introduced a suggestions list of essential subjects to report in research regarding the development of endoscopy CADx systems, aiming to facilitate more complete reporting and better comparability between studies. There is a knowledge gap regarding real-time CADx system performance during multicenter external validation. Future research should focus on development of CADx systems that can differentiate CRC from premalignant lesions, while providing an indication of invasion depth.

## Introduction

With implementation of bowel cancer screening programs, colorectal carcinoma (CRC) is increasingly being diagnosed at an early stage 1 . However, endoscopic recognition of early stage CRC is still inadequate. Community-based studies of fecal immunochemical test-positive colonoscopies show optical diagnosis sensitivities of 54.0% in a French population and 25.8% in a Dutch population for recognition of T1 CRC in nonpedunculated colorectal lesions 2 3 . Recognition of pedunculated T1 CRCs was even worse in both studies, showing sensitivities of 27.0% and 7.0%, respectively.

Classification systems such as the OPTICAL model show the potential to improve recognition of CRCs, but do not achieve satisfactory optical diagnosis performance yet and require training 4 .

Optical misdiagnosis of early CRC poses the risk of incorrect treatment. Benign and premalignant colorectal polyps can be treated safely with conventional cold or hot snare polypectomy or endoscopic mucosal resection. If the endoscopist is unable to remove a colorectal lesion en-bloc, they can proceed with a piecemeal resection technique. However, colorectal polyps that harbor early CRC should not be treated with piecemeal endoscopic resection because it is not a curative treatment for polyps that contain early CRC. In addition, dividing the colorectal lesion into multiple pieces impairs evaluation of histological high-risk factors 5 . The correct treatment for lesions suspected of presenting with early CRC is an en-bloc, organ-sparing local resection technique, such as endoscopic submucosal dissection (ESD) or endoscopic full-thickness resection (eFTR). For high-risk T1 CRC (lymphovascular invasion, high-grade budding, poorly differentiated clusters grade 2–3 or poorly differentiated histology) additional surgery is still the recommended treatment but new strategies such as adjuvant chemoradiotherapy or intensive surveillance are emerging.

Artificial intelligence (AI) can be expected to match the performance of experienced endoscopists in predicting CRC invasion depth using optical diagnosis 6 . Therefore, AI has the potential to enable local resection techniques if possible and direct referral to surgery if necessary. However, AI is not implemented in daily clinical practice for this purpose yet. Therefore, it is necessary to investigate whether use of computer-aided diagnosis (CADx) systems based on AI applied in colonoscopy results in improved distinction of early CRC from premalignant colorectal polyps.

The main goals of this review are threefold: 1) to summarize current evidence on the use of AI for improving endoscopic recognition of CRC; 2) to provide an overview of the currently used methodologies; and 3) to guide future research by exposing knowledge gaps in this upcoming field of research.

## Methods

This review was performed following the PRISMA-Scr guideline 7 .

### Search strategy

In April 2023, a literature search was conducted in the databases PubMed (including Medline), Scopus, Embase, IEEE (Institute of Electrical and Electronics Engineers) Xplore, and ACM (Association for Computing Machinery) Digital Library. The search was supervised by a medical librarian (MS). No additional sources were consulted. The search terms included the three concepts ‘colonoscopy and endoscopy’, ‘AI and computer-aided diagnosis’, as well as ‘colorectal carcinoma and colorectal polyps’. Within the three search concepts, relevant free-text terms and subject headings were paired using OR as a Boolean operator. Subsequently, the three search concepts were combined using AND as a Boolean operator. No search limits or filters were applied and no search strategies from previous reviews were used. The full search strategies and the number of records identified for each database including all terms used are presented in the Supplementary material. Email alerts were used to receive updates about new search results. In addition, the search was rerun across the databases in January 2024. The process of de-duplication was performed in EndNote according to the method described by Bramer et al. (2016) 8 .

### Study selection

Studies were considered suitable when describing use of AI for distinguishing CRC from colorectal polyps on endoscopy or endocytoscopy images and/or videos, using histopathology as the gold standard. The outcome measures sensitivity, specificity, or accuracy/area under the receiver operating curve (AUROC) had to be described. Exclusion criteria were studies solely about colorectal polyp detection, studies only describing the use of AI to improve characterization of diminutive colorectal polyps, studies without original data (reviews and letters), studies without a full-text article available, or studies unavailable in English.

Titles and abstracts of all studies identified in the primary search and rerun were screened for eligibility by two reviewers independently using the platform rayyan.ai (AT and RMS). Disagreements over the studies to be included for full-text screening were solved in a consensus meeting. Subsequently, the full texts of the remaining articles were also assessed by both reviewers independently and the final decision about which articles to include in the review was made. All cited references of included articles were checked by browsing reference lists but did not yield new inclusions. In addition, references citing the included studies were located by browsing “cited by” lists but also did not merit new inclusions.

### Data extraction

Data were extracted from the included articles by two researchers (AT and RMS) and supervised (EJS). Included studies were searched for the following data items; location and number of centers for collecting training and testing datasets; CADx system architecture; image type, magnification level, and brand; CADx system classification categories; number and type of images in training and datasets; outcome measures; key results (sensitivity, specificity, and/or accuracy/AUROC); number of endoscopists collecting data and their level of experience; and the process of image selection. No assumptions were made regarding missing information. In the case of unclear information, authors were contacted.

## Results

**Fig. 1:**
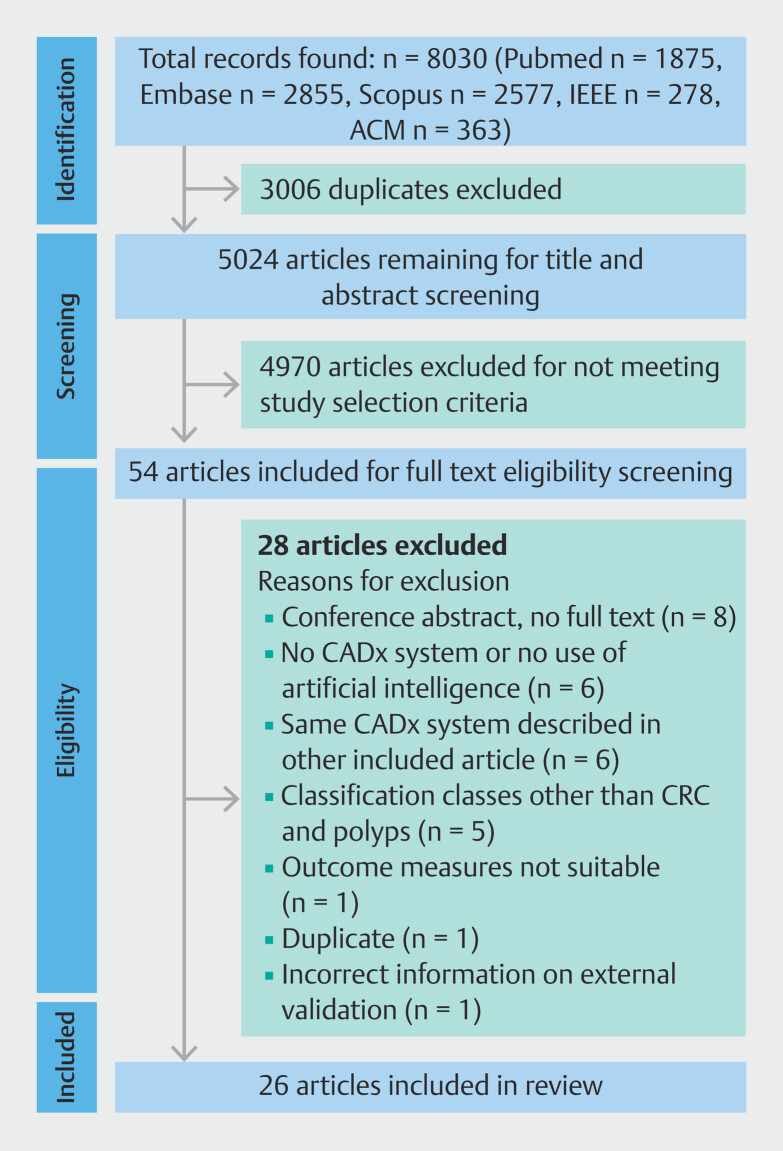
PRISMA-Scr (Preferred Reporting Items for Systematic reviews and Meta-Analyses extension for Scoping Reviews) flow chart of study selection. From: Tricco AC, Lillie E, Zarin W et al. PRISMA Extension for Scoping Reviews (PRISMA-ScR): Checklist and Explanation. Ann Intern Med 2018; 169: 467–473 doi:10.7326/M18-0850

In total, 8,030 studies were identified through the search ( Fig. 1 ). After removing duplicates, 5,024 studies remained. Based on title and abstract screening, 4,970 articles were excluded for not meeting the inclusion and exclusion criteria, leaving 54 articles. Full-text screening for eligibility resulted in exclusion of another 28 articles for various reasons. Most exclusions were conference abstracts without full text (n = 8) or were studies not describing a CADx system or not using AI at all (n = 6). Authors of the conference papers were contacted to confirm that there were no full-text articles available. Description of the same CADx system in different articles resulted in the inclusion of only one article and exclusion of the articles containing the same information (n = 6). Other reasons for full-text exclusion were classification categories other than CRC and colorectal polyps (n = 5), outcome measures not compliant with the inclusion criteria (n = 1), and a duplicate article (n = 1). Finally, one study was excluded due to incorrect information in the article regarding external validation (stating in the methods that external testing was performed while this was not actually the case), confirmed after contact with the corresponding author (n = 1). Thus, 26 articles were included in this review.

Due to heterogeneity of the CADx systems, classification categories, and datasets in all studies, a meta-analysis of the results was not appropriate. Therefore, the extracted data are presented in a qualitative manner.

### Study characteristics

An overview of the data extraction can be found in Table 1 , Table 2 , and Table 3 . With the exception of three studies, all included studies were published in the past 5 years. One study was performed in both Japan and Australia, and all other studies were solely conducted in Asian countries. One study did not mention the country where data collection took place. None of the included studies were performed real-time or as a randomized controlled trial. Most CADx systems were developed using Olympus (Tokyo, Japan) endoscopy systems (n = 23). Six CADx systems that were developed using Olympus also used Fujifilm (Tokyo, Japan) endoscopy systems. In three studies, the endoscopy brand used was not mentioned.

**Table:** **Table 1** CADx systems with CRC as separate classification category.

Authors (year)	Location, number of centers for training/testing data	CADx system architecture	Image type, magnification level and brand	Classification categories	Training data	Testing data		Outcome measures	Key results [including 95% CI if available]
Choi et al. (2021) 9	Korea, training data from one center and testing data from another center	Ensemble learning with 3 CNN models: Inception-v3, ResNet-50, and DenseNet161	White light, without magnification, Olympus	1. Normal 2. Adenoma, LGD 3. Adenoma, HGD 4. Carcinoma	N= 3,000 images (1,000 normal, 1,000 adenoma LGD, 500 adenoma HGD, 500 CRC)	N = 400 images (100 normal, 100 adenoma LGD, 100 adenoma HGD, 100 CRC)		Sensitivity, specificity, PPV, NPV	Sensitivity 77.3% Specificity 90.6%
Jheng et al. (2022) 10	Taiwan, one center for training, validation, and verification data (without cross contamination)	GUTAID system; CNN incorporating VGG16-based binary classifiers	White light, with and without magnification, Olympus	1. Normal 2. Polyp 3. Diverticulum 4. Cecum 5. Cancer	N = 9,930 images (polyp/nonpolyp 2,686/2,810, diverticulum/nondiverticulum 770/768, cecum/noncecum 339/555, cancer/noncancer 592/592, adenoma/hyperplastic polyp 409/409)	N =2,419 images (polyp/nonpolyp 672/672, diverticulum/nondiverticulum 192/192, cecum/noncecum 85/130, cancer/noncancer 148/148, adenoma/hyperplastic polyp 90/90)		Sensitivity, specificity, PPV, NPV, accuracy	*Cancer* Sensitivity 96.7% Specificity 98.3%
Mori et al. (2021) 11	Japan, training data from six centers, validation data from one center	EndoBRAIN-Plus; incorporating SVM	Endocytoscopy, Olympus	1. Invasive Cancer 2. Adenoma 3. Nonneoplastic	N = 68,082 images (image type not specified)	N = 500 ^1^ images (10 each) of 50 lesions (30 invasive cancers [8 T1, 22 T2–4], 15 adenomas [13 HGD, 2 LGD], 5 nonneoplastic)		Sensitivity and specificity for differentiating invasive cancer	Overall accuracy 91.9% [95% CI 88.8–94.3] *Differentiation cancer* Sensitivity 91.8% [95% CI 87.5–95.0] Specificity 97.3% [95% CI 93.9–99.1]
Ozawa et al. (2020) 12	Japan, one center for both training and validation data	Adapted Single Shot MultiBox Detector (SSD) network for detection/classification	White light and NBI, without magnification, Olympus	1. Adenoma 2. Hyperplastic polyp 3. SSL 4. Cancer 5. Other	N = 20,431 images (11,395 adenoma, 2,521 hyperplastic polyp, 139 SSL, 1,599 cancer, 764 other, 4,013 normal)	N = 783 white light images (582 adenoma, 125 hyperplastic polyp, 23 SSL, 29 cancer, 24 other), 290 NBI images (203 adenoma, 68 hyperplastic polyp, 6 SSL, 3 cancer, 10 other)		Accuracy, NPV, PPV	*Overall accuracy* White light 83.0% NBI 81.0%
Park et al. (2020) 13	Korea, one center for both training and testing data	Proposed CNN architecture, inspired by ResNet	Not specified	1. Normal 2. Adenoma 3. Adenocarcinoma	N = 1,848 images (449 cancer, 626 adenoma, 773 normal), after data augmentation 49,458 images	N = 410 images (128 normal, 142 adenoma, 140 cancer)		Accuracy, PPV, sensitivity	Overall accuracy 94.4% *Adenocarcinoma* Sensitivity 94.3% PPV 97.1%
Pu et al. (2020) 14	Australia/ Japan, one center for training/testing/validation data, one center for external validation data	DenseNet architecture	NBI and BLI, with magnification, Olympus and Fujifilm	Modified Sano (MS)-classification 1. MSI: hyperplastic polyp 2. MSII: LGD 3. MSIIo: SSL 4. MSIIIa: TVA/VA/HGD 5. MS IIIb: invasive cancers	N = 1,235 images (103 MSI, 429 MSII, 293 MSIIo, 295 MSIIIa, 115 MSIIIb), after data augmentation 123,500 images	N = 20 NBI images (3 MS I, 5 MS II, 2 MS IIo, 7 MS IIIa, and 3 MS IIIb) and 49 BLI images (9 MS I, 10 MS II, 10 MS IIo, 11 MS IIIa, and 9 MS IIIb)		AUROC	*Mean AUROC* NBI 84.5% BLI 90.3%
Shimizu et al. (2023) 15	Japan, one center for training and validation data	ResNet50	NBI, with and without magnification, Olympus,	1. LGD 2. HGD 3. SMs (<1000 µm) 4. SMd (>1000 µm)	N = 1,390 images (of 210 lesions; 53 LGD, 120 HGD, 20 SMs, 17 SMd) (2/3 training, 1/3 validation, repeated three times and averaged the validation results)			Accuracy (average of three-fold validation), sensitivity (image level)	*SMs* Sensitivity 96.4% Accuracy 90.7% *SMd* Sensitivity 99.2% Accuracy 92.2%
Tanwar et al. (2022) 16	Not specified, not specified	Adapted Single Shot MultiBox Detector (SSD) network for detection/classification	White light and NBI, magnification not specified, Olympus	1. Adenoma 2. Hyperplastic polyp 3. Sessile serrated lesion 4. Carcinoma 5. Other	N =20,431 images (11,395 adenomas, 2,521 hyperplastic polyps, 139 SSLs, 1,599 carcinomas, 764 other, 4,013 normal)		N = 7077 images (847 adenomas, 214 hyperplastic polyps, 41 SSLs, 33 carcinomas, 37 other, 5,905 normal)	Sensitivity, PPV, NPV, accuracy	*White light* Accuracy 83.3% *NBI* Accuracy 80.7%
Wang et al. (2023) 17	China, one center for training, validation, and test data	AFACNet; ResNet18-based architecture	White light, magnification not specified, Olympus	1. Normal 2. Polyp 3. Adenoma 4. Cancer 5. Ulcerative colitis	N= 4,591 images (865 normal, 843 polyp, 896 adenoma, 964 cancer, 1,023 ulcerative colitis) Divided 6:2:2 over training, validation, and test set			Accuracy, sensitivity, PPV	*Overall* Accuracy 89.8% Sensitivity 89.8% PPV 89.8% *Cancer* Accuracy 91.8%
Xu et al. (2023) 18	China, unspecified	ResNet101, with single shot detector (SSD)	NBI, magnification not specified, Olympus	1. Hyperplastic polyp 2. Tubular adenoma 3. Tubulovillous adenoma 4. Cancer	N = 419 images (70% training, 30% testing, type not specified)			Mean average PPV, accuracy	*Cancer* Accuracy 88.5% *Overall* Mean accuracy 74.4%
Zhou et al. (2020) 19	China, one center for training and internal testing data, two centers for external testing data	CRCNet; adapted DenseNet169 architecture	White light, without magnification, Olympus	1. Non-CRC 2. CRC (stadium I t/m IV)	N = 464,105 images (from 3,176 CRC patients and 9,003 non-CRC)	Test set 1 N = 20,783 images (from 146 CRC patients and 217 non-CRC) Test set 2 N = 15,411 images (from of 90 CRC patients and 340 non-CRC) Test set 3 N = 48,391 images (from 71 CRC patients and 1,399 non-CRC)		Accuracy, sensitivity, specificity, PPV, NPV	*Test set 1* Sensitivity 90.4% [95% CI 84.4–94.7] Specificity 85.3% [95% CI 79.8–89.7] *Test set 2* Sensitivity 78.9% [95% CI 69.0–86.8] Specificity 95.0% [95% CI 92.1–97.1] *Test set 3* Sensitivity 74.6% [95% CI 62.9–84.2] Specificity 99.2% [95% CI 98.6–99.6]
^1^ 418/500 analyzable by EndoBRAIN-Plus CADx, computer-aided diagnosis; CI, confidence interval; CNN, convolutional neural network; LGD, low-grade dysplasia; HGD, high-grade dysplasia; CRC, colorectal carcinoma; PPV, positive predictive value; NPV, negative predictive value; SVM, support vector machine; SSD, single shot detector; SSL, sessile serrated lesion; NBI, narrow band imaging; MS, modified sano; AUROC, area under the receiver operating curve; BLI, blue light imaging; TVA, tubulovillous adenoma; VA, villous adenoma; SMd, deep submucosal invasion; SMs, shallow submucosal invasion; µm, micrometer.									

**Table:** **Table 2** CADx systems with CRC invasion depth classification categories.

Authors (year)	Location, number of centers for training/testing data	CADx system architecture	Image type, magnification level and brand	Classification categories	Training data	Testing data	Outcome measures	Key results [including 95% CI if available]
Ito et al. (2018) 20	Japan, two centers for training and testing data	AlexNet	White light without magnification, Olympus	1. cTis and cT1a 2. cT1b	Group 1: N = 4,938 images (2,520 cTis and cT1a, 2,418 cT1b) and group 2: N = 5,004 (2,604 cTis and cT1a, 2,400 cT1b) After data augmentation	Number of test images before augmentation not reported. After data augmentation: N = 5,022 images (2,604 cTis and cT1a, 2,418 cT1b)	Sensitivity, specificity, accuracy, AUROC	*cTis and cT1a towards cT1b* Sensitivity 67.5% Specificity 89.0% *cT1b towards cTis and cT1a* Sensitivity 89.0% Specificity 67.5%
Minami et al. (2022) 21	Japan, two centers; training and independent validation from one and prospective test dataset from both	Unspecified CNN	White light and NBI, magnification not specified, Olympus	SMs CRC: shallow submucosal invasion (<1000 µm) SMd CRC: deep submucosal invasion (≥1000 µm)	N = 706 images (256 images of 22 SMs and 450 images of 69 SMd lesions)	N = 560 images (90 images of 9 SMs and 470 images of 47 SMd lesions)	Sensitivity, PPV, accuracy	Sensitivity 63.8%
Nakajima et al. (2020) 22	Japan, two centers for training data, one center for testing data	ResNet50	White light, without magnification, Fujinon and Olympus	Probability level for T1b diagnosis	N = 1,839 images (135 cTis, 46 T1a, 96 T1b, 37 T2) with data augmentation	N = 78 images (from 23 Tis and 21 T1b)	Specificity for T1b CRCs, sensitivity, PPV, NPV, accuracy	*Lesion-based outcome* Sensitivity 81.0% Specificity 87.0% [95% CI 66.0–97.0]
Nemoto et al. (2023) 23	Japan, ten centers, assigned to training and testing datasets (3:1)	ResNet50	White light, without magnification, Olympus and Fujifilm	cTis/T1a: noninvasion/superficial invasion T1b: deep submucosal invasion	N = 3,716 (1–10 images per lesion of 798 cTis, 100 T1a, 212 T1b)	N = 1,392 images (1–9 images per lesion of 276 cTis, 45 T1a, 82 T1b)	Specificity, sensitivity, accuracy, PPV, NPV, AUROC	*Deep submucosal invasion cancer* Sensitivity 59.8% [95% CI 48.3–70.4] Specificity 94.4% [95% CI 91.3–96.6]
CADx, computer-aided diagnosis; CI, confidence interval; cTis, carcinoma in situ; AUROC, area under the receiver operating curve; CNN, convolutional neural network; NBI, narrow band imaging; SMd, deep submucosal invasion; SMs, shallow submucosal invasion; µm, micrometer; CRC, colorectal carcinoma; PPV, positive predictive value; NPV, negative predictive value.								

**Table:** **Table 3** CADx systems with CRC in a classification category together with adenomas.

Authors (year)	Location, number of centers for training/testing data	CADx system architecture	Image type, magnification level and brand	Classification categories	Training data	Testing data	Outcome measures	Key results [including 95% CI if available]
Katayama et al. (2023) 24	Japan, two centers; training dataset from one center and testing dataset from another center	CADx, ResNet34	NBI, with and without magnification, Olympus	1. JNET 1 2. JNET 2a 3. JNET 2b 4. JNET 3	N = 5,610 images (949 JNET 1, 1,023 JNET 2a, 1,097 JNET 2b, 970 JNET 3, 1,571 normal mucosa)	N = 480 images (one in each magnification mode of 160 cases; 24 JNET 1 [2 hyperplastic polyp, 22 SSL], 81 JNET 2A [78 adenoma, 3 Tis], 40 JNET 2B [11 adenoma, 20 Tis, 9 T1b], 15 JNET 3 [T1b])	Sensitivity, specificity, PPV, NPV, accuracy	*Type 2B* Sensitivity 60.9% Specificity 84.3% Accuracy 80.9% *Type 3* Sensitivity 64.6% Specificity 98.9% Accuracy 93.8%
Lu, Y et al (2022) 25	China, three centers used for training and internal validation data	CAD-N; ResNeSt-based architecture	NBI, without magnification, Olympus	1. Hyperplastic and inflammatory polyps 2. Adenomatous polyps, intramucosal cancer, superficial submucosal invasive cancer 3. Deep submucosal invasive cancer 4. Normal mucosa	N = 8,458 images (1,983 type 1, 3,063 type 2, 591 type 3, 2,821 type 4)	N =2,115 images (513 type 1, 739 type 2, 143 type 3, 720 type 4)	Sensitivity, specificity, PPV, NPV, accuracy	Sensitivity 92.0% [95% CI 90.5–93.4] Specificity 94.9% [95% CI 93.0–96.3]
Lu, Z et al. (2022) 26	China, training and internal test datasets from one center and external test dataset from two centers	Endo-CRC; Resnet50-based architecture	White light and NBI/BLI, with and without magnification, Olympus and Fujifilm	1. Suit-ER: suitable for endoscopic resection (LGD, HGD, intramucosal cancer, CRC with SMI <1000 µm) 2. Unsuit-ER: unsuitable for endoscopic resection (CRC with submucosal invasion ≥1000 µm and advanced CRC)	N = 3,334 white light images and 4,580 NBI/BLI images of 268 suit-ER lesions and 1,388 white light and 3,493 NBI/BLI images of 82 unsuit-ER lesions	External validation N = 211 white light images and 540 NBI/BLI images of 49 suit-ER lesions and 22 white light and 65 NBI/BLI images of 6 unsuit-ER lesions	Sensitivity, specificity, PPV, NPV, accuracy	*Image pairs (NBI/BLI and white light)* Sensitivity 78.4% Specificity 89.2%
Lui et al. (2019) 27	China, one center for training and independent test set	CNN architecture with ResNet50 backbone	White light and NBI, without magnification, Olympus	1. Endoscopically curable lesion (SSL, adenoma, slightly invasive adenocarcinoma [SM1]) 2. Endoscopically incurable lesion (invasive adenocarcinoma [SM2])	N = 8,000 images (4,000 images of 1,159 endoscopically curable lesions and 4,000 images of 493 endoscopically incurable lesions)	N = 567 images (of 10 SSLs, 28 tubular adenomas, 8 tubulovillous adenomas, 10 SM1 CRCs, 20 SM2 CRCs)	AUROC, sensitivity, specificity, PPV, NPV, accuracy	*Predicting endoscopically curable lesions* Sensitivity 88.2% [95% CI 84.7–91.1] Specificity 77.9% [95% CI 70.3–84.4]
Luo et al. (2021) 28	China, one center for training data and independent test set	AEWL; GoogLeNet-based architecture	White light, without magnification, brand not specified	1. P0: noninvasion or superficial submucosal invasion 2. P1: deep invasion	N= 7,734 images (424 P0 and 233 P1 lesions) (data augmentation applied)	N= 1,634 images (81 P0 and 75 P1 lesions)	AUROC, sensitivity, specificity, PPV, NPV, accuracy	*P0 vs P1* Sensitivity 91.2 [95% CI 88.8–93.3] Specificity 91.0 [95% CI 89.0–92.7] *P0 vs P1 (excluding advanced CRC)* Sensitivity 65.3 [95% CI 61.9–68.8] Specificity 68.5 [95% CI 63.9–73.1]
Okamoto et al. (2021) 29	Japan, one center for training data and independent test set	CADx-N (NICE classification) and CADx-J (JNET classification); ResNet18 models	NBI, with and without magnification, Olympus	*CADx-N* NICE type 1 NICE type 2 NICE type 3 *CADx-J* JNET type 1 JNET type 2A JNET type 2B JNET type 3	*CADx-N* N = 4,156 NBI images (of 409 resected and 227 nonresected lesions) *CADx-J* N = 3,670 images (without magnification excluded)	N = 480 images (of 2 hyperplastic polyps, 22 SSLs, 89 LGD, 23 HGD, 24 deep submucosal invasive carcinoma), of which 320 with magnification	Specificity, sensitivity, accuracy, PPV, NPV	*CADx-N NICE 3* Sensitivity 61.1% [95% CI 55.3–63.1] Specificity 99.5% [95% CI 98.5–99.9] *CADx-J JNET 3* Sensitivity 62.5% [95% CI 55.2–64.2] Specificity 99.6% [95% CI 98.3–99.9]
Song et al. (2020) 30	Korea, one center for training data and two independent test sets	ResNet50 and DenseNet201	NBI, with magnification, Olympus	1. Serrated polyps 2. Benign conventional adenomas/mucosal or superficial submucosal cancer 3. Deep submucosal cancers	N = 624 images (48 hyperplastic polyps, 76 SSLs, 393 adenomas, 62 mucosal or superficial submucosal tumor, 45 deep submucosal cancers)	N test set I = 182 images (15 hyperplastic polyps, 24 SSLs, 106 adenomas, 20 mucosal or superficial submucosal tumor, 17 deep submucosal cancers) N test set II = 363 images (30 hyperplastic polyps, 70 SSLs, 206 adenomas, 28 mucosal or superficial submucosal tumor, 29 deep submucosal cancers)	Cohen’s kappa (Agreement between true and predicted histopathology), accuracy, sensitivity, specificity, PPV and NPV	*Benign conventional adenomas/mucosal or superficial* *Test set I* Sensitivity 84.1% Specificity 75.0% *Test set II* Sensitivity 88.5% Specificity 72.1% *Deep submucosal cancers* *Test set I* Sensitivity 58.8% Specificity 93.3% *Test set II* Sensitivity 62.1% Specificity 96.7%
Takemura et al. (2012) 31	Japan, one center for training and validation data	HuPAS version 3.1; SVM classifier	NBI, with magnification, Olympus	1. Hiroshima type A 2. Hiroshima type B-C3	N = 1,519 images (of 454 type A and 1,065 type B-C3 lesions)	N = 47 Hiroshima type A and 324 Hiroshima type B-C3	Sensitivity, specificity	Sensitivity 97.8% Specificity 97.9%
Tamai et al. (2017) 32	Japan, one center for testing data, training data not specified	Not specified	NBI, with magnification, Olympus	Sano’s colorectal M-NBI classification A: hyperplastic polyps B: adenomas, CRC with invasion depth <1000 µm C: CRC with invasion depth ≥1000 µm	Not specified	N = 121 images (of 21 hyperplastic polyps, 80 adenomas/ CRC with invasion depth <1,000 µm, 20 CRC with invasion depth ≥1,000 µm)	Sensitivity, specificity, PPV, NPV, accuracy	*Neoplastic lesions* Sensitivity 95.0% Specificity 100% *Deep SMI lesions* Sensitivity 55.0% Specificity 95.0%
Tokunaga et al. (2020) 33	Japan, two centers for both training and testing data	Adapted Single Shot MultiBox Detector (SSD) with MobileNets as baseline	White light, without magnification, Olympus and Fujifilm	1. Endoscopically treatable (LGD, HGD, CRCs with SMI <1000 µm) 2. Endoscopically untreatable (CRCs with SMI ≥1000 µm, advanced CRCs)	N = 2,751 images (353 LGD, 1,259 HGD, 333 CRCs with SMI <1,000 µm, 328 CRCs with SMI ≥1,000 µm, 478 advanced CRCs)	N = 691 images (88 LGD, 316 HGD, 83 CRC with SMI <1,000 µm, 84 CRCs with SMI ≥1,000 µm, 120 advanced CRCs	Sensitivity, specificity, PPV, NPV, accuracy, average time for diagnosis	Sensitivity 96.7% Specificity 75.0%
Yang et al. (2020) 34	Korea, three centers used for training and test dataset (divided at a ratio 9:1 using random sampling in each lesion category) and external validation data from another center	ResNet152 and Inception-ResNet-v2	White light, without magnification, Olympus en Fujinon	1. Advanced CRC (T2-T4) 2. Early CRC/HGD 3. Tubular adenoma 4. Nonneoplastic lesions	N = 3,453 images (813 nonneoplasms, 1,187 TA, 557 HGD, 168 T1, 125 T2, 534 T3, 69 T4)	External validation N= 240 images (3 advanced CRC, 8 early CRC/HGD, 116 TA, 113 nonneoplastic)	Discrimination performance for seven- and four category classification	*7-class performance* Mean accuracy 74.7% [95% CI 73.3–76.2] *4-class performance* Mean accuracy 79.2% [95% CI 76.5–81.8]
CADx, computer-aided diagnosis; CI, confidence interval; NBI, narrow band imaging; JNET, Japan NBI expert team; PPV, positive predictive value; NPV, negative predictive value; BLI, blue light imaging; SMI, submucosal invasion; µm, micrometer; ER, endoscopic resection; LGD, low-grade dysplasia; HGD, high-grade dysplasia; CRC, colorectal carcinoma; SSL, sessile serrated lesion; AUROC, area under the receiver operating curve; NICE, narrow band imaging international colorectal endoscopic; SVM, support vector machine; SSD, single shot detector; TA, tubular adenoma.								

One CADx system was developed for endocytoscopy. The CADx systems developed for conventional endoscopy were mainly trained and tested with white light images only (n = 10). In some cases, the image enhancement techniques narrow band imaging (NBI) and/or blue light imaging (BLI) were used (n = 9), or both NBI/BLI and white light were used (n = 5). One study did not report the imaging mode.

Conventional endoscopy images used for CADx training and testing were mostly nonmagnified (n = 11). Several studies used both magnified and nonmagnified images (n = 5), or magnified images only (n = 4). Five studies did not report whether or not magnification was used.

### Classification categories

A large variation was seen in the classification categories used. The number of classification categories varied between two and five. In eleven studies, the CADx system was trained to diagnose CRC in a separate classification category ( Table 1 ). Eleven different studies reported on CADx systems that classified CRCs together with adenomas ( Table 3 ). In four studies, CADx systems were trained with the purpose to estimate the CRC invasion depth ( Table 2 ).

**Fig. 2:**
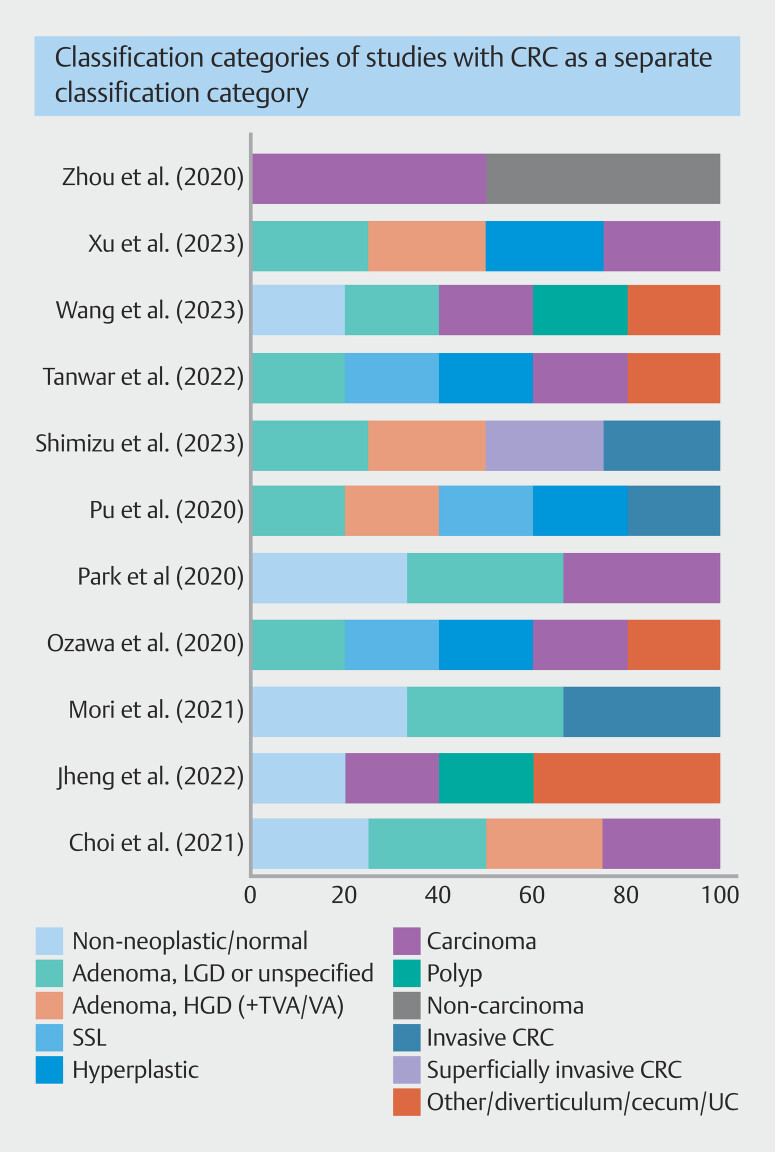
Overview of the number and type of classification categories used in studies with CADx systems trained to classify colorectal carcinoma as a separate classification category.

In the studies that classified CRC as a separate category ( Table 1 ), the other categories were diverticulum, cecum, or other, nonneoplastic or normal, hyperplastic polyp, sessile serrated lesion, polyp, adenoma with low-grade dysplasia or unspecified, adenoma with high-grade dysplasia, noncarcinoma, carcinoma, or advanced CRC or invasive cancer. In one study, adenoma with high-grade dysplasia contained (tubulo-)villous adenomas as well. The number and type of classification categories for the CADx systems that classified CRC as a separate category can be seen in Fig. 2 .

The category types were partially comparable in the second group of studies ( Table 3 ), except for the main difference that early CRC was classified together with benign or premalignant colorectal lesions. Of these 11 studies, five used dichotomous categories such as endoscopically treatable (including adenomas) or endoscopically untreatable, adenoma or invasive cancer, noninvasion/superficial invasion or deep invasion, and Hiroshima A or Hiroshima B-C3. The remaining six studies in this group either added a third or fourth category such as normal/nonneoplasms, serrated, or hyperplastic polyps (n = 4) or used the NICE and JNET classification (n = 2).

The studies about CADx systems trained to estimate CRC invasion depth reported different, mostly dichotomous categories based on the possibility of endoscopic resection ( Table 2 ): cTis/cT1a (in situ or high-grade dysplasia/slightly invasive) or cT1b (deeply invasive), shallow submucosal invasion (< 1,000 µm) or deep submucosal invasion (≥ 1,000 µm), and Tis/T1a (noninvasion/superficial invasion) or T1b (deep submucosal invasion). In contrast to the previous group, the classification categories of these CADx systems did not include benign or premalignant colorectal lesions. In one case, the CADx system provided a probability level for the diagnosis CRC stage T1b.

### CADx system architecture

Articles detailing the algorithm employed either utilize convolutional neural networks (CNN) (n = 19) or leverage a more conventional machine learning approach, using support vector machines (SVM) (n = 2). Most CNN-based algorithms use various ResNet or DenseNet architectures (n = 16) as their backbone, except for one study using AlexNet, one study using GoogLeNet, and one study using multiple binary classifiers. One of the CNN-based studies used ensemble learning with three CNN models. In three studies, single shot detector (SSD) methods were used. Two studies did not specify their CADx system architectures. With the exception of one study using EndoBRAIN, no studies reported use of a regulatory-approved CADx system.

### Training and testing datasets

The majority of studies collected endoscopic images from one center (n = 12), using separate datasets for training and testing. Studies that collected data from two centers kept the data separate for a training and testing dataset (n = 2) or mixed the data from both centers in their training and testing dataset (n = 3). In addition, there were four studies that gathered data in three centers, either using one center for training and internal test data, and two centers for external testing (n = 2), using two centers for training data and one for testing data (n =1), or combining the data from all three centers for training and validation (n = 1). Finally, the remaining centers (n = 3) reported data collection in a larger number of centers, namely four (three for training/testing data and one for validation), seven (six for training and one for validation), and 10 (all data together divided 3:1 over training/testing). Two studies did not specify the origin of their image datasets.

Information regarding how many endoscopists created the images used for CADx training and testing and the level of experience of these endoscopists at the time is unclear in many studies. Only four studies mentioned a level of experience of the endoscopists capturing the images used in their datasets, stating that the endoscopists were either certified or experienced (n = 2), images were captured by several endoscopists including trainees (n = 1) or that three endoscopists with at least 5 years of colonoscopy experience took the images (n = 1). Regarding image selection, most studies reported that images were selected based on quality or region of interest, and reported that this selection took place subjectively by endoscopists or one of the authors (n = 11). Other studies reported either nothing about image quality checks (n = 7) or merely that image quality was checked but not by whom (n = 7). One study specified that no inclusion or exclusion criteria regarding image quality were applied. Of the 18 studies that reported image selection, 14 studies elaborated on the type of quality assessment. This mostly entailed the exclusion of images that were out of focus, blurred, too bright, or containing blood, feces, or mucus.

**Fig. 3:**
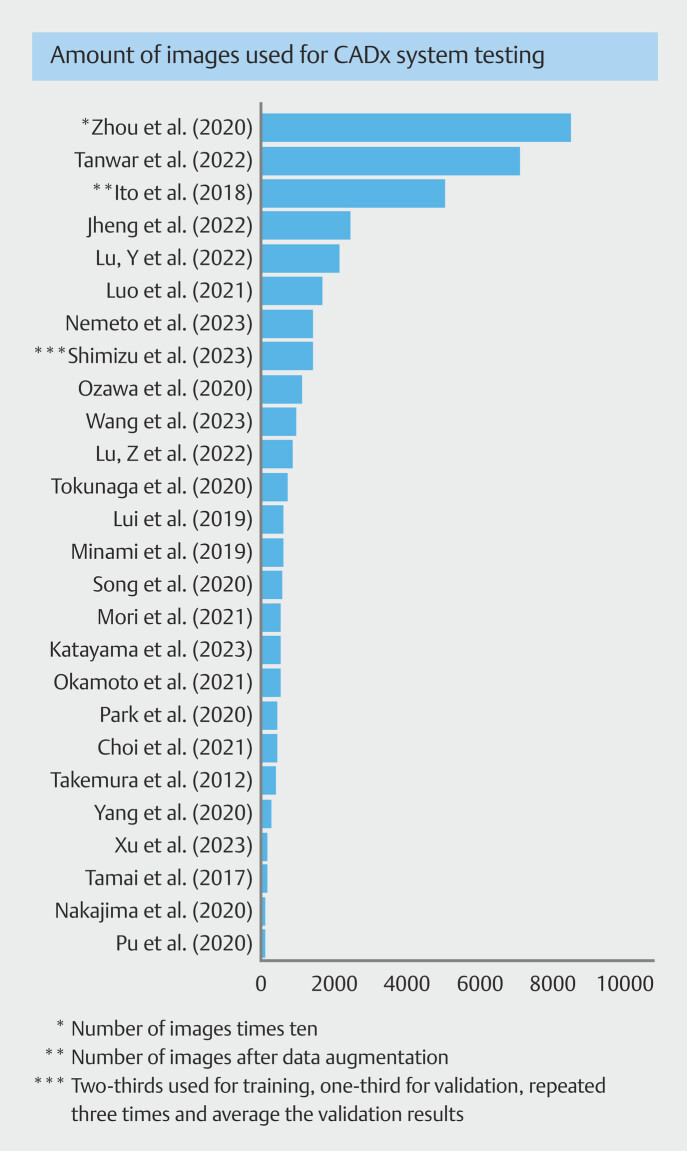
Bar graph illustrating the number of images used in computer-aided diagnosis system testing datasets.

The number of images used in testing databases for the CADx systems varied from 69 to 84,585, the latter using dozens of images from one lesion ( Fig. 3 ). Databases of several hundred images were most common (121–918, n = 15). Over 1,000 images were used for testing in nine studies (ranging between 1,073–7,077 in eight studies and 84,585 divided over three test sets in one study), whereas less than 100 images were used in two studies (69 and 78 images). In the training databases, the differences between studies were even greater, with the number of images varying between 293 and 464,105. Although many studies used multiple images from one lesion and possibly multiple lesions from one patient, only three studies reported use of clustering/bootstrap sampling.

### Diagnostic performance

The diagnostic performance of all CADx systems should be interpreted in the context of the methodologies used for testing ( Table 1 , Table 2 , Table 3 ). Calculation of the diagnostic performance was based on prediction of the CADx system defined as correct when the classification category was compliant with the histopathology result. Diagnostic performance was divergent, with sensitivities varying from 55.0% to 99.2%, specificities from 67.5% to 100%, and accuracies from 74.4% to 94.4%.

## Discussion

This review provides an overview of current research on use of AI to improve endoscopic recognition of early stage CRC and indicates important directions for future research on this topic.

Application of AI to improve endoscopic recognition of early stage CRC is a recently growing research field, almost solely in Asian countries. Compared with other applications of AI in gastroenterologic health care, such as in Barrett’s esophagus, or for detection of colorectal polyps, AI for early stage CRC is in its infancy.

Although promising diagnostic performance of CADx systems are reported in current literature, many studies lack the proper methodology necessary to rely on translation of these results to daily clinical practice. In addition, this review demonstrates that the information necessary for interpreting the results is presented inconsistently, often resulting in missing crucial details. Therefore, we compiled a list of recommended essential subjects that should be reported in future research regarding development of CADx systems to improve endoscopic recognition of gastrointestinal diseases. This list was created by consensus among all authors with both clinical and technical backgrounds, based on the findings from this review. With this list, we aim to facilitate more complete reporting in future research and better comparability between studies.

Recommended essential subjects to describe in research regarding the development of endoscopic computer-aided diagnosis systems to improve quality and comparability:

- Data collection
  - In which center(s) did data collection take place?
  - Was there separate data collected for training and testing?
  - How many endoscopists collected data?
  - How many images were acquired per patient? (range)
  - Were the data acquired in different endoscopy rooms?
  - Did image/video selection based on quality take place?
  - Which criteria were used to assess image/video quality?
- Data type
  - From which endoscopy brand is the data?
  - Do the training and testing datasets contain magnified images?
  - Do the training and testing datasets contain virtual chromoendoscopy images?
  - Do the images/videos used contain the entire lesion or a proportion of the lesion?
- Training/testing databases
  - Was a power calculation performed for the number of images used for computer-aided diagnosis system validation?
  - How many images were used in the training and testing datasets?
  - What is the distribution of lesion types and number of lesions?
  - Was data augmentation applied?
  - Was an external test employed?
  - How were the images distributed over training/validation/test sets?
- Computer-aided diagnosis (CADx) system properties
  - Which classification categories does the CADx system use?
  - Is the CADx system output specified? (e.g. calibrated confidence score 0–1 for all classes)
  - What is the CADx system input (single frame or video)?
  - Is pre-processing applied (e.g. quality selection)?
- Computer-aided diagnosis system testing
  - Did real-time testing take place?
  - What was the level of experience of endoscopists who performed testing?
- Outcome measures
  - How well does the computer-aided diagnosis system perform on the clinical outcome measures sensitivity, specificity, negative predictive value, positive predictive value, and diagnostic accuracy?

Large variation in the classification categories used for CADx systems included in this review complicates a head-to-head comparison of diagnostic performance. In addition, multiple studies reported on CADx systems with classification categories that do not contribute to a solution for existing clinical challenges. For example, high diagnostic performance in a CADx system that distinguishes invasive CRC from adenomas 11 or in some cases even the cecum, diverticula, and normal tissue 10 , will most likely not improve optical diagnosis because these distinctions are already quite easy to make for most endoscopists.

AI may offer a solution to three relevant issues for optical diagnosis of colorectal polyps and CRC. First, improving optical diagnosis of diminutive benign and premalignant polyps can enable implementation of “diagnose and leave” and “resect and discard” strategies 35 . Second, improving differentiation between premalignant polyps and early stage CRC can prevent unjustly piecemeal resections and subsequent segmental colon resection. Finally, improving the estimation of invasion depth ensures en-bloc local resection if possible and surgical resection if necessary. The choice of CADx system classification categories determines whether these issues can be resolved. This review shows that a high number of CADx systems (11 of 26 studies, 42%) that classify early CRC together with adenomas. Therefore, future research in this field should focus on development of CADx systems with classification categories compliant with at least one of the problems stated here. We recommend developing CADx systems able to first distinguish CRC from benign and premalignant colorectal polyps. Subsequently, the CADx system should be able to estimate invasion depth. Categories relevant to clinical practice should be used for this, such as the Kikuchi levels combined with a category ≥ T2 CRC.

Using magnifying chromoendoscopy or virtual chromoendoscopy such as NBI shows improvement in optical diagnosis of endoscopists for T1 CRC 36 and is already specifically recommended for optical diagnosis of early gastric cancer 37 . This suggests that using virtual chromoendoscopy might also be relevant to incorporate in CADx systems for early CRC, and the same applies to using magnification. With only five of the CADx systems for conventional endoscopy in this review using both white light and BLI/NBI images, an advantage can be seen regarding use of image enhancement techniques.

In this review, one CADx system for CRC based on endocytoscopy was found 11 . Endocytoscopy enables in vivo microscopic assessment of a colorectal lesion. With correct interpretation, this should certainly improve endoscopic recognition of early CRCs. However, endocytoscopy is not widely available and requires intensive training. Endoscopic recognition of early CRC needs to be improved in all endoscopy centers, especially nonexpert centers that might be less likely to offer endocytoscopy. AI for endoscopy has the potential to be widely available. Therefore, research should focus on CADx systems for conventional endoscopy. Another limitation of endocytoscopy seems to be that a relatively high number of images cannot be analyzed by the CADx system EndoBRAIN-plus (82 out of 500 images 11 ).

A notable finding of this review is that many studies gathered training and testing data from the same center. External validation and specifically geographic validation (on data collected by independent researchers at different centers) is important to verify CADx system generalizability and to confirm that overfitting does not occur 38 39 . For example, Nemoto et al. 23 collected data in a large consortium of 10 centers. However, all data together were divided over a training and testing dataset with a 3:1 ratio, also known as split-sample validation 38 . This addresses internal validity but does not provide information on generalizability of CADx system performance. Furthermore, data splitting is generally considered an unstable validation method when the number of subjects is low (i.e., less than 20,000) 40 . Keeping data separate for external validation, while paying attention to a useful number of images from each classification category, seems to be a more suitable use of the data available. During external validation, CADx systems can also be tested on multiple endoscopy brands. Only six studies reported on the use of data from multiple endoscopy brands to train/test their CADx system. Future studies should try to incorporate testing on multiple endoscopy brands into their CADx systems to ensure widespread usability.

It is important to point out, with regard to CADx system generalizability, that there are large differences in the number of images for both training and testing databases. Small training databases might decrease robustness of a CADx system. If possible, power calculations should be performed to determine the appropriate sample size for CADx system validation. For example, the method performed by Rondonotti et al. (2022) can be used 41 .

In addition, this review showed that CADx systems for recognition of early CRC have not been tested in real time during endoscopy procedures. Only with real-time testing can the value of a CADx system in clinical practice be determined 38 . CADx systems need to provide fast characterizations in order to be a valuable addition to clinical practice. Another advantage of real-time testing is that the CADx diagnosis can easily be compared with the endoscopist’s optical diagnosis. This comparison is necessary to verify whether the CADx system is indeed able to improve the endoscopist’s optical diagnosis.

Furthermore, still images in databases collected for CADx system development are often high-quality images. This is confirmed in this review, with many studies that select their data based on image quality. Creating high-quality images during endoscopy can be challenging, especially for less experienced endoscopists. Previous research has shown that factors such as inadequate lighting, motion blur, and compression artifacts can introduce significant challenges for CADx systems. For instance, a study by Jaspers et al. 42 provides insights into how image degradation can cause a decline in CADx system performance, even though the images may still appear realistic from a human visual perspective. Therefore, it is crucial to account for potential image degradation during development and training of these systems to ensure reliable performance in real-time clinical practice.

Moreover, use of still images raises the question whether the entire lesion was visualized properly or only a part of the lesion, particularly because lesions containing CRC are often larger and difficult to visualize in one frame. Preferably, a standardized image selection protocol should be used in order to prevent a CADx system from being trained or tested with an image that does not contain the cancerous part of a lesion but is labeled with the gold standard histopathology result of CRC.

Finally, the CADx architecture explored in this study represents a range of approaches from traditional machine learning to state-of-the-art deep learning models. In two studies 11 43 , SVMs were used. SVM is a traditional machine learning algorithm effective in high-dimensional spaces, versatile in handling various classification problems, and relatively easy to interpret. However, its training can be slow, especially with large datasets. Most studies use CNNs, a class of deep neural networks widely used for image analysis due to their ability to automatically and adaptively learn spatial hierarchies of features from input images. The studies reviewed employed various types of CNN architecture, differing in the number of parameters, layers, and overall complexity, each designed to address specific challenges. Each study developed or utilized a CNN-based CAD system tailored to optimize performance for their specific dataset, underscoring the adaptability and robustness of CNNs in diverse image analysis tasks.

This review has several limitations. First, study quality was not taken into account due to the qualitative approach of this review. This was because, given the heterogeneity of CADx system architecture (most important among other things), classification categories, and testing data, pooled results of CADx system performance would lead to a distorted picture. Second, bias can occur in the data represented for data extraction, although we attempted to prevent this by independent data extraction with references to where the data was extracted from in the papers.

## Conclusions

In conclusion, this review provides a present-day overview of research regarding CADx systems for endoscopic recognition of early CRC. Future research should focus on relevant classification categories and multicenter external validation with sufficient original test data, preferably in real time. The list of recommended essential subjects to describe in research regarding development of endoscopic computer-aided diagnosis systems is a useful tool for future research in this field.

## Supplementary Material

Supplementary Material Zusatzmaterial
