# Supplementary material for: Improving the endoscopic recognition of early colorectal carcinoma using artificial intelligence: current evidence and future directions
Source: Endosc Int Open. 2024 Oct 10;12(10):E1102–17. doi: 10.1055/a-2403-3103 (PMC11466514; doi:10.1055/a-2403-3103)
Supplement: Supplementary file 1 — Supplementary Material [file 10-1055-a-2403-3103_24053918.pdf.pdf]

Supplementary material

Supplementary material

Supplementary Table 1 IEEE search 09.01.2024.

Full search	((((IEEE Terms:endoscopes) OR (Abstract:"endoscop*") OR (Abstract:"colonoscop*"))) AND ((IEEE Terms:Computational and artificial intelligence) OR (IEEE Terms:Computer aided diagnosis) OR (IEEE Terms:Classification algorithms) OR (Abstract:"artificial intelligence") OR (Abstract:"AI") OR (Abstract:"neural network") OR (Abstract:"deep learning") OR (Abstract:"machine learning") OR (Abstract:"computer aided") OR (Abstract:"computer assisted") OR (Abstract:"CADx"))) AND ((IEEE Terms:tumors) OR (IEEE Terms:neoplasms) OR (IEEE Terms:cancer) OR (Abstract:"neoplas*") OR (Abstract:"tumor") OR (Abstract:"adenoma*") OR (Abstract:"carcinoma*") OR (Abstract:"malignan*") OR (Abstract:"polyp*") OR (Abstract:"lesion*") OR (Abstract:"CRC"))	378
Context	("IEEE Terms":tumors) OR ("IEEE Terms":neoplasms) OR ("IEEE Terms":cancer) OR ("Abstract":"neoplas*") OR ("Abstract":"tumor") OR ("Abstract":"adenoma*") OR ("Abstract":"carcinoma*") OR ("Abstract":"malignan*") OR ("Abstract":"polyp*") OR ("Abstract":"lesion*") OR ("Abstract":"CRC")	49,082
Concept	("IEEE Terms":Computational and artificial intelligence) OR ("IEEE Terms":Computer aided diagnosis) OR ("IEEE Terms":Classification algorithms) OR ("Abstract":"artificial intelligence") OR ("Abstract":"AI") OR ("Abstract":"neural network") OR ("Abstract":"deep learning") OR ("Abstract":"machine learning") OR ("Abstract":"computer aided") OR ("Abstract":"computer assisted") OR ("Abstract":"CADx")	381,475
Population	("IEEE Terms":endoscopes) OR ("Abstract":"endoscop*") OR ("Abstract":"colonoscop*")	5,203

Supplementary material

Supplementary Table 2 ACM guide to computing literature search 09.01.2024.

Full search	(Title:(colo* AND (neoplas* OR tumor* OR tumour* OR adenoma* OR carcinoma* OR malignan* OR cancer* OR polyp* OR lesion*) OR CRC) OR Abstract:(colo* AND (neoplas* OR tumor* OR tumour* OR adenoma* OR carcinoma OR malignan* OR cancer* OR polyp* OR lesion*) OR CRC)) AND (Title:("AI" OR "artificial intelligence" OR "neural network" OR "CNN" OR "DNN" OR "deep learning" OR "machine learning" OR "computer assisted" OR "computer aided" OR "CADx") OR Abstract:("AI" OR "artificial intelligence" OR "neural network" OR "CNN" OR "DNN" OR "deep learning" OR "machine learning" OR "computer assisted" OR "computer aided" OR "CADx")) AND (Title:(endoscop* OR colonoscop*) OR Abstract:(endoscop* OR colonoscop*))	247
Context	Title:(colo* AND (neoplas* OR tumor* OR tumour* OR adenoma* OR carcinoma* OR malignan* OR cancer* OR polyp* OR lesion*) OR CRC) OR Abstract:(colo* AND (neoplas* OR tumor* OR tumour* OR adenoma* OR carcinoma OR malignan* OR cancer* OR polyp* OR lesion*) OR CRC)	11,856
Concept	Title:("AI" OR "artificial intelligence" OR "neural network" OR "CNN" OR "DNN" OR "deep learning" OR "machine learning" OR "computer assisted" OR "computer aided" OR "CADx") OR Abstract:("AI" OR "artificial intelligence" OR "neural network" OR "CNN" OR "DNN" OR "deep learning" OR "machine learning" OR "computer assisted" OR "computer aided" OR "CADx")	326,671
Population	Title:(endoscop* OR colonoscop*) OR Abstract:(endoscop* OR colonoscop*)	2,373

Supplementary material

Supplementary Table 3 Scopus search 09.01.2024.

Full search	(TITLE-ABS-KEY ("AI" OR "artificial intelligence" OR "neural network" OR "CNN" OR "DNN" OR "deep learning" OR "machine learning" OR "computer assisted diagnos*" OR "computer aided diagnos*" OR "computer aided characteri*" OR "computer assisted characteri*" OR "computer aided classification" OR "computer assisted classification" OR "CADx")) AND (TITLE-ABS-KEY ("endoscop*" OR "colonoscop*")) AND (TITLE-ABS-KEY ("CRC" OR ("colo*" AND ("neoplas*" OR "tumor*" OR "tumour*" OR "adenoma*" OR "carcinoma*" OR "malignan*" OR "cancer*" OR "polyp*" OR "lesion*"))))	2,571
Context	"CRC" OR ("colo*" AND ("neoplas*" OR "tumor*" OR "tumour*" OR "adenoma*" OR "carcinoma*" OR "malignan*" OR "cancer*" OR "polyp*" OR "lesion*")) *(search within 'Article title, Abstract, Keywords')*	756,421
Concept	"AI" OR "artificial intelligence" OR "neural network" OR "CNN" OR "DNN" OR "deep learning" OR "machine learning" OR "computer assisted diagnos*" OR "computer aided diagnos*" OR "computer aided characteri*" OR "computer assisted characteri*" OR "computer aided classification" OR "computer assisted classification" OR "CADx"<br>*(search within 'Article title, Abstract, Keywords')*	2,039,875
Population	"endoscop*" OR "colonoscop*"<br>*(search within 'Article title, Abstract, Keywords')*	504,432

Supplementary material

Supplementary Table 4 Pubmed search 09.01.2024.

Full search	("endoscopy"[MeSH Terms] OR ("endoscop*"[Title/Abstract] OR "colonoscop*"[Title/Abstract])) AND ("artificial intelligence"[MeSH Terms] OR "diagnosis, computer assisted"[MeSH Terms] OR ("AI"[Title/Abstract] OR "artificial intelligence"[Title/Abstract] OR "neural network"[Title/Abstract] OR "CNN"[Title/Abstract] OR "DNN"[Title/Abstract] OR "deep learning"[Title/Abstract] OR "machine learning"[Title/Abstract] OR "computer assisted diagnos*"[Title/Abstract] OR "computer aided diagnos*"[Title/Abstract] OR "computer aided characteri*"[Title/Abstract] OR "computer assisted characteri*"[Title/Abstract] OR "computer aided classification"[Title/Abstract] OR "computer assisted classification"[Title/Abstract] OR "CADx"[Title/Abstract])) AND ("colorectal neoplasms"[MeSH Terms] OR "colonic polyps"[MeSH Terms] OR ("colo*"[Title/Abstract] AND ("neoplasms"[MeSH Terms] OR "adenoma"[MeSH Terms] OR "hyperplasia"[MeSH Terms] OR "carcinoma"[MeSH Terms])) OR (("colo*"[Title/Abstract] AND ("neoplas*"[Title/Abstract] OR "tumor*"[Title/Abstract] OR "tumour*"[Title/Abstract] OR "adenoma*"[Title/Abstract] OR "carcinoma*"[Title/Abstract] OR "malignan*"[Title/Abstract] OR "cancer*"[Title/Abstract] OR "polyp*"[Title/Abstract] OR "lesion*"[Title/Abstract])) OR "CRC"[Title/Abstract]))	1,847

Supplementary material

Supplementary Table 5 Embase search 09.01.2024.

Full search	(exp colorectal tumor/ or exp colorectal polyp/ or (("colo*" and ("neoplas*" or "tumor*" or "tumour*" or "adenoma*" or "carcinoma*" or "malignan*" or "cancer*" or "polyp*" or "lesion*")) or "CRC").ti,ab,kf) and (exp machine learning/ or exp artificial intelligence/ or computer assisted diagnosis/ or ("AI" or "artificial intelligence" or "neural network" or "CNN" or "DNN" or "deep learning" or "machine learning" or "computer assisted diagnos*" or "computer aided diagnos*" or "computer aided characteri*" or "computer assisted characteri*" or "computer aided classification" or "computer assisted classification" or "CADx").ti,ab,kf) and (exp colonoscopy/ or exp endoscopy/ or ("endoscop*" or "colonoscop*").ti,ab,kf)	2,749
Context	exp colorectal tumor/ or exp colorectal polyp/ or (("colo*" and ("neoplas*" or "tumor*" or "tumour*" or "adenoma*" or "carcinoma*" or "malignan*" or "cancer*" or "polyp*" or "lesion*")) or "CRC").ti,ab,kf.	1,190,515
Concept	exp machine learning/ or exp artificial intelligence/ or computer assisted diagnosis/ or ("AI" or "artificial intelligence" or "neural network" or "CNN" or "DNN" or "deep learning" or "machine learning" or "computer assisted diagnos*" or "computer aided diagnos*" or "computer aided characteri*" or "computer assisted characteri*" or "computer aided classification" or "computer assisted classification" or "CADx").ti,ab,kf.	846,131
Population	exp colonoscopy/ or exp endoscopy/ or ("endoscop*" or "colonoscop*").ti,ab,kf	1,471,904
